# Performance Improvement of Partial Least Squares Regression Soluble Solid Content Prediction Model Based on Adjusting Distance between Light Source and Spectral Sensor according to Apple Size

**DOI:** 10.3390/s24020316

**Published:** 2024-01-05

**Authors:** Doo-Jin Song, Seung-Woo Chun, Min-Jee Kim, Soo-Hwan Park, Chi-Kook Ahn, Changyeun Mo

**Affiliations:** 1 Department of Interdisciplinary Program in Smart Agriculture, Kangwon National University, Chuncheon-si 24341, Republic of Korea; doojin0463@kangwon.ac.kr (D.-J.S.); weez96@kangwon.ac.kr (S.-W.C.); tnghks1357@kangwon.ac.kr (S.-H.P.); 2 Agriculture and Life Sciences Research Institute, Kangwon National University, Chuncheon-si 24341, Republic of Korea; kim91618@kangwon.ac.kr; 3 Korea Agriculture Technology Promotion Agency, Iksan-si 54667, Republic of Korea; ahnck@koat.or.kr; 4 Department of Biosystems Engineering, Kangwon National University, Chuncheon-si 24341, Republic of Korea

**Keywords:** apple size, soluble solid content, visible near-infrared spectroscopy, partial least squares regression, optimal distance

## Abstract

Apples are widely cultivated in the Republic of Korea and are preferred by consumers for their sweetness. Soluble solid content (SSC) is measured non-destructively using near-infrared (NIR) spectroscopy; however, the SSC measurement error increases with the change in apple size since the distance between the light source and the near-infrared sensor is fixed. In this study, spectral characteristics caused by the differences in apple size were investigated. An optimal SSC prediction model applying partial least squares regression (PLSR) to three measurement conditions based on apple size was developed. The three optimal measurement conditions under which the Vis/NIR spectrum is less affected by six apple size levels (Levels I–VI) were selected. The distance from the apple center to the light source and that to the sensor were 125 and 75 mm (Distance 1), 123 and 75 mm (Distance 2), and 135 and 80 mm (Distance 3). The PLSR model applying multiplicative scatter correction pretreatment under Distance 3 measurement conditions showed the best performance for Level IV-sized apples ($R_{pre}^{2}$ = 0.91, RMSEP = 0.508 °Brix). This study shows the possibility of improving the SSC prediction performance of apples by adjusting the distance between the light source and the NIR sensor according to fruit size.

## 1. Introduction

In 2020, fruit consumers in the Republic of Korea prioritized fruit quality over price to a greater degree than that in 2018 [1,2]. Apples are the most cultivated fruit among the country’s representative fruits and are popular among consumers [3]. Currently, apple importation is banned in the country. Thus, selecting high-quality local apples is crucial because of the inevitable future competition with imported apples [4,5].

The criteria for selecting apples can be broadly divided into external and internal qualities. The external quality can be classified into size, color, weight, shape, and external defects, whereas the internal quality can be classified into sugar content, acidity, moisture content, and internal defects [6]. Various studies have measured the internal quality of fruits, such as the soluble solid content (SSC), using near-infrared (NIR) spectroscopy. NIR spectroscopy can quickly and non-destructively determine and sort the internal quality of fruits [7].

NIR spectroscopy is a technique that measures transmitted or reflected light when an NIR sensor with a wavelength range of 700–2500 nm is applied to fruits, measuring SSC through partial least squares regression (PLSR) [8]. Although multiple linear regression (MLR) and principal component regression (PCR) have been used as SSC prediction models, PLSR has been widely used. PLSR is a method for finding latent variants to effectively describe concentration changes using both concentration and spectral data from samples, allowing multiple response variables to be simultaneously modeled while effectively handling multicollinearity and noisy independent variables [9]. The application of NIR spectroscopy to fruit quality analysis involves a reflectance mode that uses reflected light for irradiated light, a full transmittance mode that uses transmitted light inside the fruit, and a semi-transmittance mode that uses only a part of the fruit [10]. Recently, the full transmittance mode has been used to sort the SSC of fruits, and the mode speedily measures the overall SSC of fruits. However, the full transmittance mode varies in the spectrum because of the changes in the path length or scattering caused by differences in the sample size [11]. This variation reduces the accuracy of the SSC predictions [12]. Therefore, studies have been conducted to improve the accuracy of PLSR models for fruit SSC prediction, primarily by performing spectral preprocessing, and to reduce this disturbance [13].

In developing an SSC prediction model, Suh et al. [10] found that the internal reflectance mode and transmittance mode (90°, 180°) are excellent for pear spectroscopy, and the coefficient of determination of the cross-validation ($R_{cv}^{2}$) and root mean square error of prediction (RMSEP) of the PLSR model without pretreatment were 0.777 and 0.38 °Brix, 0.643 and 0.48 °Brix, respectively. The RMSEP of the multiplicative scatter correction (MSC) pretreatment application model showed that the internal reflectance mode was 0.37–0.57 °Brix, and the transmittance mode (90°) was 0.39–0.51 °Brix. Shin [14] investigated the SSC prediction of melons using NIR spectroscopy. Among the various pretreatments performed, range normalization is the pretreatment with the best prediction performance, with an $R_{cv}^{2}$ of 0.755 and RMSEP of 0.89 °Brix. Luo et al. [15] developed a sugar prediction PLSR model using three wavelength bands and five pretreatments of navel oranges. The model with standard normal variate (SNV) pretreatment in the wavelength band of 450–1800 nm showed optimal performance with $R_{v}^{2}$ of 0.8514 and RMSE of 1.1649. Kawano et al. [12] non-destructively measured the SSC of satsuma mandarins using an NIR transmittance spectrum, in which the wavelength affected by the fruit diameter was 844 nm. After applying a second-order differential to a value of 844 nm and normalizing the result, the PLSR analysis resulted in an R of 0.989 and SEP of 0.32. Tian et al. [16] investigated the optimal apple SSC prediction through spectroscopic analysis using Vis/near-infrared (Vis/NIR) and pretreatment applications. PLSR was used for model development, and a total of 322 apples were used: the correlation coefficient of the cross-validation (R_cv_) was 0.8545, and the root mean square error of the cross-validation (RMSECV) was 0.5730 without pretreatment. Optimal preprocessing was performed with mean normalization and 11-point smoothing, where the correlation coefficient of prediction (R_pre_) was 0.8744 and RMSEP was 0.5332.

Although previous studies have improved the accuracy of the SSC prediction model by applying spectral preprocessing, changes in the optical path corresponding to changes in the size of the fruit have not been considered because the location of the light source and NIR sensor are fixed when measuring the spectrum of the fruit. Because the sizes of fruits vary and the difference in diameter for each sample is large, the change in the NIR spectral signal also considerably influences the outcome. This phenomenon reduces the accuracy of fruit SSC prediction during high-speed sorting. Therefore, it is necessary to determine the distance between the optimal light source and the NIR sensor for each fruit size.

This study aimed to determine the optimal distance between the light source and NIR sensor based on the apple size for predicting apple’s SSC and develop a PLSR model for predicting SSC for apple size based on the determined distance. Particularly, the spectral distance characteristics between the light source and the NIR sensor were analyzed based on the apple size, and various forms of spectral preprocessing were applied.

## 2. Materials and Methods

In this study, the first experiment (Experiment 1) aimed to select the optimal distance between the light sources and sensors that had less influence on apple size, while the second experiment (Experiment 2) aimed to develop a PLSR SSC prediction model for each apple size at the selected optimal distance.

### 2.1. Experimental Samples

The apple of the Fuji cultivar (Malus pumila) used in this experiment was purchased from the Chungju Agricultural Products Processing Center (APC), and its size was classified using the Korean Agricultural Product Standard Notice (Table 1). The weight, diameter, and height of all samples were measured after purchase, and the average weight, diameter, and height of the apples are listed in Table 2. The apples were stored in a refrigerator at 4 °C, and tempering was performed in a laboratory at 20 ± 1 °C for more than 5 h to reduce the effect of the temperature before the spectral signal measurement experiment.

The samples in Experiment 1 were classified from Levels I to V according to weight, and three apples for each level were used. The average weights were 389.75, 324.62, 291.9, 240.22, and 197.14 g, in descending order of magnitude.

The samples in Experiment 2 were classified from Levels I to VI according to their weights, and 82, 57, 72, 60, 70, and 70 apples from Levels I to VI were used. The average weights were 398.02, 318.41, 258.16, 223.19, 194.55, and 182.28 g, from the largest to the smallest.

### 2.2. Spectra Collection and SSC Measurement

**Figure 1:**
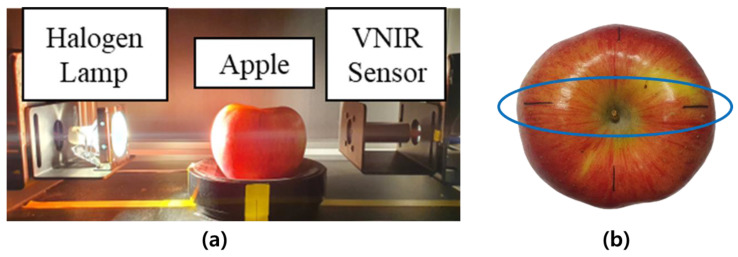
(**a**) Vis/NIR spectrum measurement system for apples and (**b**) mark of maximum diameter of apple.

An NIR spectroscopy device was used to measure the Vis/NIR spectra, and it consisted of a light source, a sample fixing part, and a spectral sensor, as shown in Figure 1a. A 12 V, 100 W tungsten–halogen lamp was used as the light source, and the light source was placed at the equatorial position of the apple. The spectral sensor was connected to a spectrometer (USB4000; Ocean Optics, Dunedin, FL, USA) via a fiber optic cable. Spectroscopic measurements were performed after 1 h of light stabilization.

In Experiment 1, the spectra were measured at an integration time of 200 ms, and the values of ten measurements were averaged. The point corresponding to the maximum diameter of the apple was measured three times by allowing light to penetrate, and then an average spectrum of the three measurements was produced. In Experiment 2, the spectra were measured at an integration time of 100 ms, and the values of five measurements were averaged. The spectra were measured once in four directions (0°, 90°, 180°, 270°) according to the maximum diameter (0°) of the apple, and the average spectrum in the four directions was used. For rapid measurement considering the application of an online system, the integration time and average time were differently set from Experiment 1, and the spectrum was measured in four directions to reflect the influence of the size of the apple and the measurement area of the apple.

After measuring the spectra, four directions of the spectrum-measured apple were cut to make nectar using a mixer, and the juice was extracted using a filter. A refractometer (PAL-3; ATAGO, Tokyo, Japan) was used to measure the SSC in four directions per apple. The resolution of the refractometer used was 0.1 °Brix at 0.1 °C, and the accuracy was ±0.1 °Brix.

The measured spectrum was configured at intervals of approximately 0.2 nm with a wavelength of 470 to 1150 nm. The light source and Vis/NIR sensor were placed at the center of the height of the apple in all experiments.

### 2.3. Analysis of Spectral Characteristics and Selection of Appropriate Distance between Light Source and Vis/NIR Sensor (Experiment 1)

**Figure 2:**
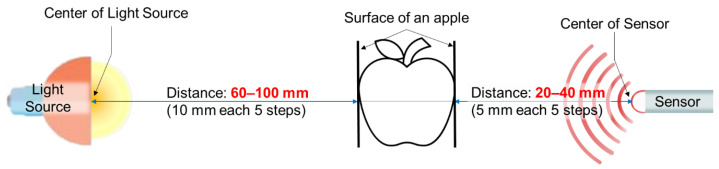
Design of distance between light source, apple, and NIR sensor for NIR signal acquisition.

In Experiment 1, the spectral characteristics were investigated based on the distance between the apple surface, light source, and Vis/NIR sensor. As the size of the apple changed, the distance between the apple surface, light source, and Vis/NIR sensor varied. The spectral signal was measured by changing the position of the light source and Vis/NIR sensor around the apple to determine the characteristics of the spectrum that occurred as this distance changed. The light source was measured at distances of 60, 70, 80, 90, and 100 mm from the apple surface (Figure 2). When the light source was closer than 60 mm, the apple was burned, and the spectral signal was weak when it was farther than 100 mm; therefore, these distances were excluded. Vis/NIR sensors were used for measurements at distances of 20, 25, 30, 35, and 40 mm from the apple surface. When the distance of the sensor was less than 20 mm from the surface of the smallest apple, the large apple collided with the Vis/NIR sensor, and the spectral signal weakened when it exceeded 40 mm; therefore, these distances were excluded.

The intensity of light with a wavelength of 714.17 nm, mainly representing the maximum value among the various wavelengths, was used to analyze the tendency of the distance change between the light source and Vis/NIR sensor in the measured spectra. For the intensity of light measured by the distance between the light source and the Vis/NIR sensor, the coefficient of variation (CV) was used to obtain the distance range of the light source and the Vis/NIR sensor that was the least affected by the distance change between the light source and the Vis/NIR sensor. The CV is a unitless constant that represents the degree of variation with respect to the mean of the population; the lower it is, the more uniform it is. CV is the standard deviation (SD) divided by the mean, as expressed in Equation (1).

(1) $$CV=\frac{SD}{Mean}$$

**Figure 3:**
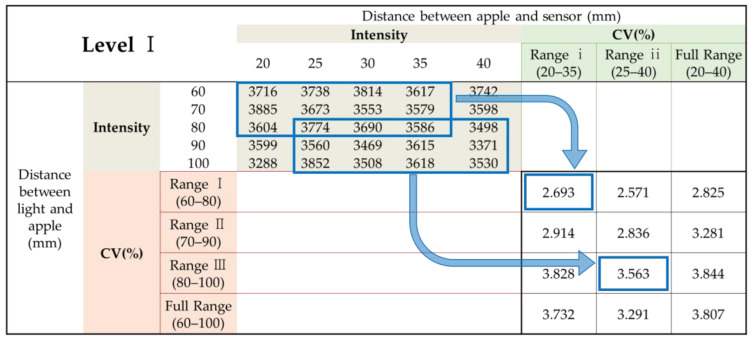
The 714.17-nm transmittance intensity value of apples and CV (%) calculation method for each distance range (Level I apples).

The distance range between the light source and the Vis/NIR sensor was defined to include all sizes of apples used in the experiment. The maximum diameter difference between the largest and smallest apples used was approximately 30 mm (radius difference of approximately 15 mm); therefore, the light source and the Vis/NIR sensors were defined at 20 and 15 mm intervals, respectively (Table 3). Figure 3 illustrates the CV calculations for each section. After averaging the CV for each section calculated for each level, the three lowest values were selected as the appropriate distance between the light source and the Vis/NIR sensor.

### 2.4. Development of Apple SSC Prediction Model (Experiment 2)

The PLSR model was applied to develop an optimal SSC prediction model for each apple size at an appropriate distance between the light source and the Vis/NIR sensor selected in Section 2.3. The calibration model for SSC prediction and the calibration dataset (Prediction) were randomly divided into a 7:3 ratio for verification. The model was applied to each of the three distances for each apple size, and the spectrum was measured in four directions for each apple, resulting in the number of spectra within the dataset being four times the number of apples. A calibration model for SSC prediction was developed using 70% of the calibration dataset. Cross-validation was performed, and the performance was verified by applying the remaining 30% of the unknown verification dataset. Equation (2) is used in the PLSR model and is given by

(2) $$X=TP^{T}+E,\;Y=UQ^{T}+F,\;U=TB+H$$

where *X* is an independent variable (spectral matrix); *U* is a score matrix that describes the dependent variable *Y*; P is an eigenvalue matrix of the independent variable; Q is an eigenvalue matrix of the dependent variable; E, F, and H are residual matrices; and *B* is a regression coefficient of PLSR [17]. Unscrambler X (v10.4; CAMO SOFTWARE AS, Oslo, Norway) was used to construct the PLSR model.

- Spectral preprocessing

In spectroscopic analysis, noise is caused by changes in the optical path because of the size of the fruit, reflected and scattered light, and changes in the state of the spectroscopic equipment. In this experiment, to reduce this effect, the location of the light source and the sensor were adjusted to determine the appropriate distance; however, we minimized the noise by preprocessing the spectrum. The maximum normalization, range normalization, mean normalization, standard normal variate (SNV), and MSC methods were used for preprocessing, which was performed using the Unscrambler X 10.4 software.

- Model evaluation

The performance of the SSC prediction model was evaluated using the coefficient of determination of calibration ($R_{cal}^{2}$), coefficient of determination of prediction ($R_{pre}^{2}$), RMSEC, and RMSEP. Each metric is expressed as follows:

$$R_{cal}^{2}=1-\frac{\sum_{i=1}^{n_{c}}{\left(y_{mi}-y_{pi}\right)}^{2}}{\sum_{i=1}^{n_{c}}{\left(y_{mi}-y_{mean}\right)}^{2}},$$

$$R_{pre}^{2}=1-\frac{\sum_{i=1}^{n_{p}}{\left(y_{mi}-y_{pi}\right)}^{2}}{\sum_{i=1}^{n_{p}}{\left(y_{mi}-y_{mean}\right)}^{2}},$$

$$RMSEC=\sqrt{\frac{1}{n_{c}}\sum_{i=1}^{n_{c}}{\left(y_{pi}-y_{mi}\right)}^{2},}$$

$$RMSEP=\sqrt{\frac{1}{n_{p}}\sum_{i=1}^{n_{p}}{\left(y_{pi}-y_{mi}\right)}^{2}},$$

where $y_{pi}$ and $y_{mi}$ are the predicted and measured SSC of the *i*th apple, respectively, and $y_{mean}$ is the average value of the calibration set or prediction set. $n_{c}$ and $n_{p}$ are the numbers of apples in the calibration and prediction sets, respectively. The closer the $R_{cal}^{2}$ and $R_{pre}^{2}$ values are to 1, the lower the RMSEC and RMSEP values. The smaller the difference, the better the model.

## 3. Results and Discussion

### 3.1. Transmittance Spectral Characteristics According to Apple Size and Light Source and Vis/NIR Sensor Distance (Experiment 1)

**Figure 4:**
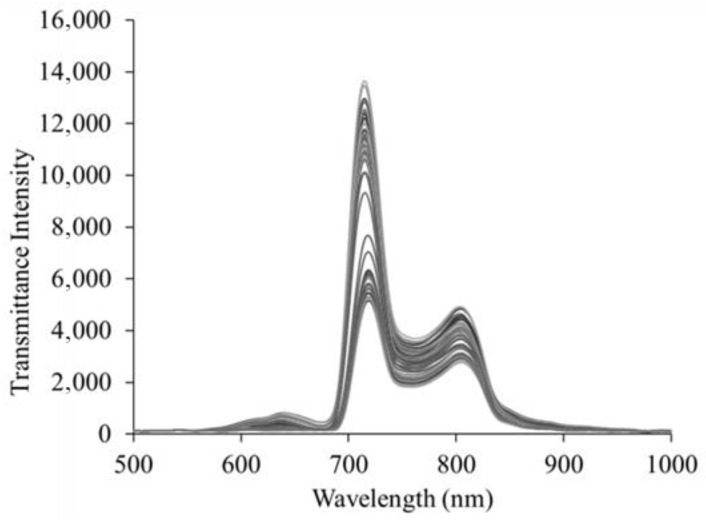
Vis/NIR spectra of Level IV apple samples.

The transmittance spectrum was measured at five distances between apples and light sources for five levels (Levels I–V) of apple samples and five distances between apples and Vis/NIR sensors. Figure 4 shows the transmittance spectrum of apples corresponding to Level IV, indicating large absorption rates in the ranges of 640–700 nm and 700–900 nm. Among the wavelengths corresponding to the visible light region, the absorption peak at approximately 675 nm is related to pigment compounds, such as anthocyanin and chlorophyll (a, b), in apple peel [18]. Moreover, absorption peaks at approximately 660, 745, and 840 nm are associated with the third overtone of carotenoids and O-H stretching [19]. Wavelengths of approximately 750, 850, and 895 nm are associated with the third overtone of C-H and H_2_O [20]. O-H and C-H bonds have been reported to be associated with SSC [21].

**Figure 5:**
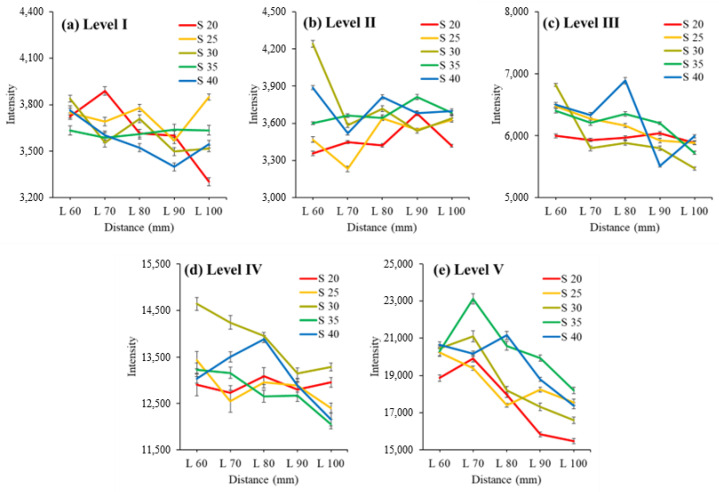
Transmittance intensity of 714 nm for each distance between light source and apple surface for distance between the apple surface and NIR sensor. Note: S: distance between apple surface and NIR sensor (mm); L: distance between light source and apple surface (mm).

**Figure 6:**
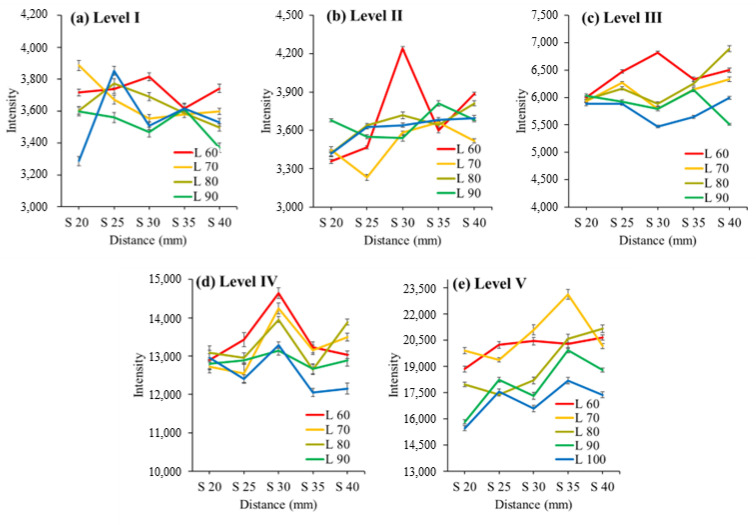
Transmittance intensity of 714 nm for each distance between apple surface and NIR sensor for distance between light source and apple surface. Note: S: distance between apple surface and sensor (mm); L: distance between light source and apple surface (mm).

**Figure 7:**
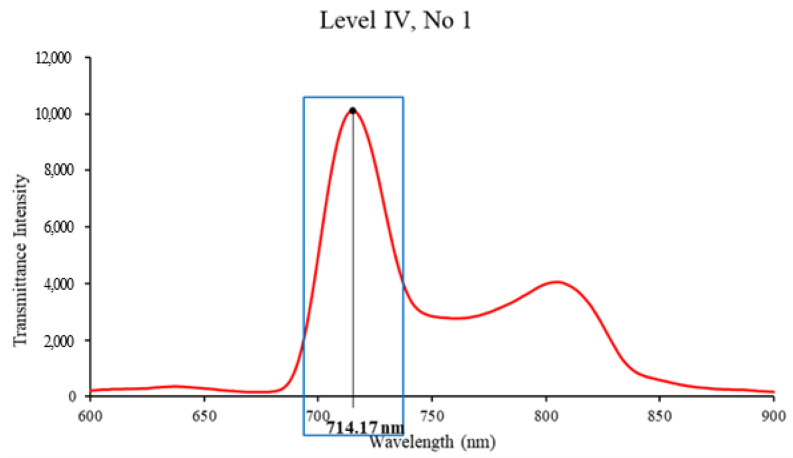
Selection of the highest-intensity wavelength in the apples’ spectra.

After measuring the transmittance spectra of three apples of size Levels I to V, the average spectrum was calculated for each level. Figure 5 and Figure 6 show the transmittance intensity (Figure 7) at a wavelength of 714.17 nm, representing the maximum value in the average spectrum for each apple level. Figure 5 illustrates the transmittance intensity against the distance between the light source and the apple surface based on the distance between the apple surface and Vis/NIR sensor. Figure 6 shows the transmittance intensity against the distance between the apple surface and Vis/NIR sensor based on the distance between the light source and the apple surface.

The intensity of the NIR signal decreased as the distance between the light source and the apple increased, as shown in Figure 5. In addition, the difference in the intensity values of the NIR signal was larger as the distance between the light source and the apple increased compared with that of the apples of other levels in Level V, the smallest apple (Level V: 15.2%, Levels I–IV: 2.6–10.0%).

As shown in Figure 6, the intensity of light increased at Levels II, III, and V as the distance between the apple surface and the NIR sensor increased. In addition, the difference in signal strength value was larger as the distance between the apple surface and the near-infrared sensor increased compared with that of the apples of other levels in Level V, which was the smallest apple (Level V: 11.8%, Levels I–IV: 1.5–7.3%).

Hence, the smaller the size of the apple, the greater the distance between the apple and the light source, and the greater the distance between the apple and Vis/NIR sensor, the greater the influence on the transmittance signal. In addition, the transmittance signal decreased as the light source moved farther away, whereas as the Vis/NIR sensor moved farther away, the transmittance signal increased. Thus, a difference in the transmittance signal appeared when the distance between the light source and the Vis/NIR sensor changed, which we determined would affect the SSC measurement of the apple.

### 3.2. Selection of Appropriate Distance between Light Source and Vis/NIR Sensor According to Changes in Apple Size

The measured intensity value was divided by the distance range of the light source and the Vis/NIR sensor, as shown in Table 3, to determine the appropriate distance between the light source and the Vis/NIR sensor based on changes in the apple size. The CV was calculated for each level. Table 4 shows the CV for each level calculated for each distance range as an average value, and the ranking is based on the low value of the CV. As shown in Table 4, when the distance range of the light source or Vis/NIR sensor was used as a whole, most results were poor with the highest CV. The wider the distance range in which the light source or Vis/NIR sensor was located, the greater the deviation of the measured spectral signals. In addition, regardless of the distance range of the Vis/NIR sensor, the CV was low in the order of distance ranges III, I, and I of the light source. This phenomenon occurred possibly because the closer the light source was to the apple, the greater the difference in the amount of transmitted light was. However, regardless of the distance range of the light source, no clear tendency was observed in the distance range of the Vis/NIR sensor. This result is observed because the transmittance spectrum was more affected by the distance range of the light source than by that of the Vis/NIR sensor. The lowest CV appeared when the distance between the light source and the apple surface was 80–100 mm and the distance range between the apple surface and the Vis/NIR sensor was 20–35 mm. That is, the corresponding distance range had the least change in the transmittance signal, even when the distance between the light source and the Vis/NIR sensor changed because of the change in the size of the apple. In addition, three distance ranges were selected as appropriate distances between light sources and Vis/NIR sensors with a CV (%) of less than 5 (distance range of 80–100 mm between the light source and apple surface and 25–40 mm between the apple surface and the Vis/NIR sensor, distance range of 70–90 mm between the light source and the apple surface and 20–35 mm between the apple surface and the Vis/NIR sensor).

**Figure 8:**
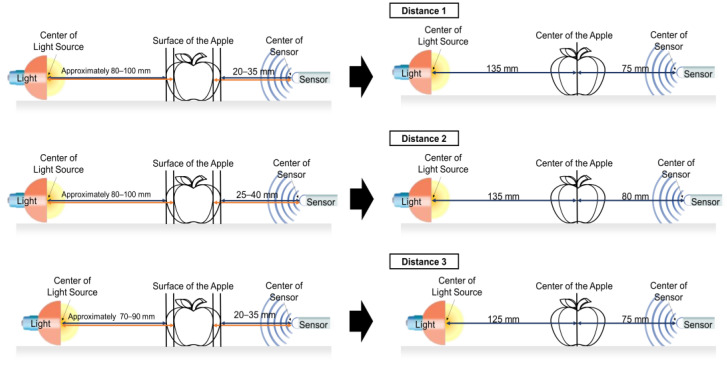
Converting distance range of light source–apple–sensor to fixed distances.

The appropriate distance between the selected light source and Vis/NIR sensor is the range of distance from the apple surface, and the position of the light source and Vis/NIR sensor changes relative to the apple size changes. The distance measurement criteria of the light source and Vis/NIR sensor were converted from the apple surface to the apple center. The distance between the light source and the Vis/NIR sensor was converted to a fixed distance by adding the radius of the largest apple (55 mm) among the samples to the minimum distance range such that most apples were in each distance range regardless of size (Figure 8). Therefore, the appropriate distance between the selected light source and the Vis/NIR sensor is 135 mm for the light source and 75 mm for the sensor, 135 mm for the light source and 80 mm for the sensor, and 125 mm for the light source and 75 mm for Distances 1, 2, and 3, respectively.

### 3.3. Characteristics According to Size of Apple Sample (Experiment 2)

**Figure 9:**
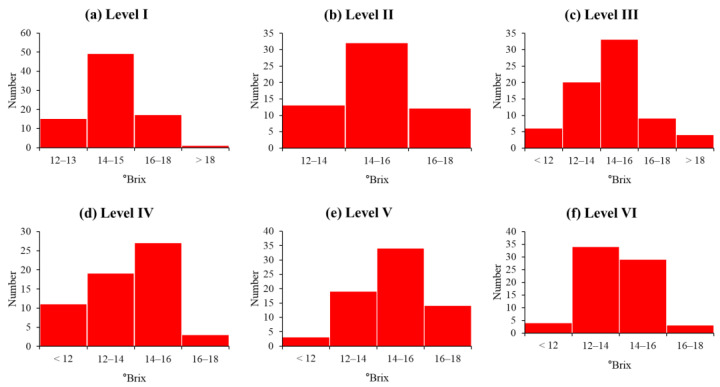
SSC distribution of apple samples by each level.

Figure 9 illustrates the SSC distribution of the 411 apples used in the development of SSC prediction models by apple size according to the appropriate distance (Experiment 2). Table 5 lists the number of apples used in the calibration model and those used in the prediction model, and the mean and SD of SSC by the apple level. The number of apples used in the model of calibration and prediction was randomly divided by 7:3. Additionally, the spectrum was measured in four directions with 0-degree rotation of the maximum diameter of the apples at 0°, 90°, 180°, and 270°; thus, the spectrum was measured as many times as the number of apples multiplied by 4. The table also presents the number of spectra. From Levels I to VI, the number of apples used in the model construction was 58, 40, 51, 42, 49, and 49, and the number of apples used in the prediction model was 24, 17, 21, 18, 21, and 21. The SSC ranges from Levels I to VI were 12.05–18.80 °Brix, 12.93–16.80 °Brix, 10.08–18.68 °Brix, 9.38–16.85 °Brix, 10.25–17.90 °Brix, and 10.70–16.45 °Brix, respectively. The SD of SSC from Levels I to VI was distributed as 1.06–1.80. 

### 3.4. Development of SSC Prediction Model Based on Distance between Light Source and Vis/NIR Sensor

An SSC prediction model for each apple size was developed using three appropriate distances from the light source and the previously identified Vis/NIR sensor. The three appropriate distances used resulted in the smallest changes in the transmittance spectrum despite changes in the size of the apple. The SSC prediction PLSR model was developed by measuring the transmittance spectrum at the corresponding distance for each level to confirm the effect of apple size (i.e., the three appropriate distances for each level) on the apple SSC prediction performance.

**Figure 10:**
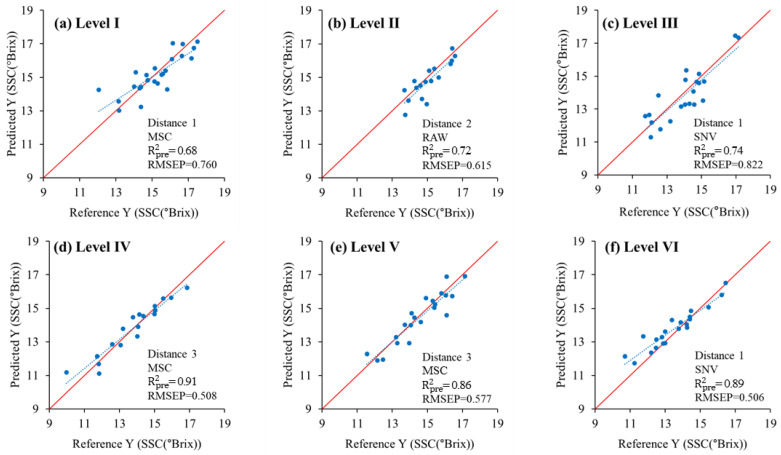
Results of validating best SSC prediction models for apple size with unknown samples.

**Figure 11:**
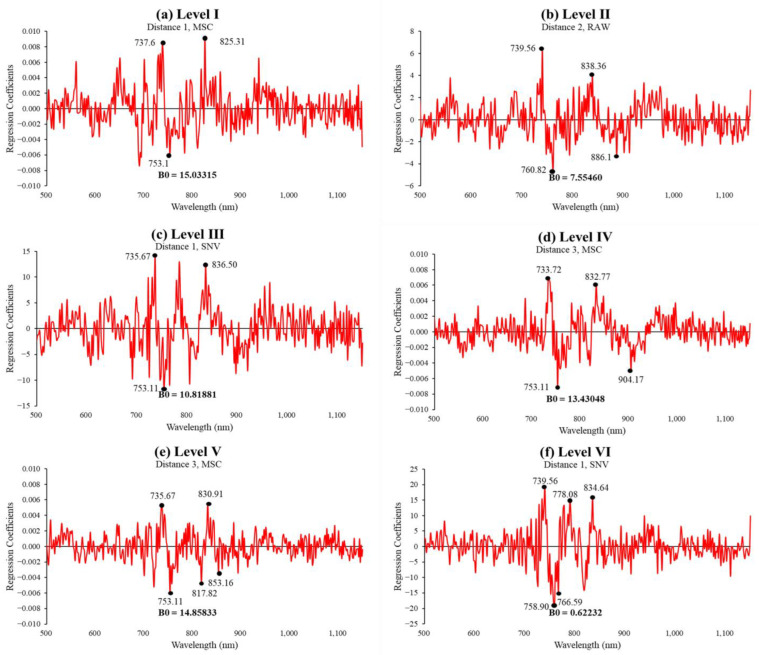
Regression coefficient of the best SSC prediction models for apple size with unknown samples.

Transmittance spectra were measured in four directions (i.e., 0°, 90°, 180°, and 270°) according to the area showing the maximum diameter of each apple. The average spectrum measured in the four directions for each apple was used as the spectrum for each apple. The apple SSC prediction models were developed by applying each of the eight preprocessing types. The performance of the developed models was verified using unknown samples. Table 6, Table 7, Table 8, Table 9, Table 10 and Table 11 compare the results of the SSC prediction model developed for Levels I–VI by the apple size and the model performance with preprocessing applied, showing the best performance for each selected distance. Figure 10 and Figure 11 show the results and regression coefficient of the model that performed best among the three distances. Figure 11 shows that each model has a relatively large correlation at wavelengths of 745, 850, and 895 nm related to sugar content. During preprocessing, the Savitzky–Golay first/second-order derivatives showed extremely low performance and are not shown in the table.

For Level I, the SSC prediction model, which measured the transmittance spectrum at Distance 1 among the three distance conditions and applied MSC pretreatment, showed the best performance (Table 6). The $R_{cal}^{2}$ and RMSEC of the calibration model of this predictive model were 0.9 and 0.414, respectively, and $R_{pre}^{2}$ and RMSEP were 0.68 and 0.769 °Brix, respectively, as verified using unknown samples (Figure 10a). The performance of the SSC prediction model was excellent in the order of Distances 2 and 3, and the optimal preprocessing conditions occurred when the SNV was applied.

In Level II, among the three distance conditions, the PLSR SSC prediction model without spectral preprocessing performed the best under Distance 2 conditions (Table 7). The $R_{cal}^{2}$ and RMSEC of its calibration model were 0.96 and 0.223, and the factor was 13. In predicting using unknown samples, $R_{pre}^{2}$ and RMSEP were 0.72 and 0.615 °Brix, respectively (Figure 10b). The performance of the SSC prediction model was excellent in the order of Distances 1 and 3, and in all cases, preprocessing was not applied.

For Level III, the SSC prediction model exhibited the best performance under the Distance 1 conditions (Table 8). When SNV preprocessing was applied, $R_{cal}^{2}$ and RMSEC of the calibration model were 0.99 and 0.142, respectively, and the factor was 15. In predicting with unknown samples, $R_{pre}^{2}$ and RMSEP were 0.74 and 0.822 °Brix, respectively (Figure 10c). The performance of the SSC prediction model was excellent in the order of Distances 2 and 3, and the optimal preprocessing methods were mean normalization and SNV, respectively.

In Level IV, the PLSR SSC prediction model that applied MSC preprocessing under Distance 3 conditions had the highest prediction accuracy (Table 9). The $R_{cal}^{2}$ and RMSEC of the model were 0.99 and 0.195, and the factor was 12. In predicting with unknown samples, $R_{pre}^{2}$ and RMSEP were 0.91 and 0.508 °Brix, respectively, showing the best performance among all levels, as shown in Figure 10d. The performance of the SSC prediction model was excellent in the order of Distances 1 and 2, and the optimal preprocessing was maximum normalization and range normalization.

In Level V, the SSC prediction model that applied MSC preprocessing at Distance 3 among the three distance conditions showed the best performance, as shown in Table 10. For this SSC prediction model, $R_{cal}^{2}$ and RMSEC of the calibration model were 0.90 and 0.487, and in predicting with unknown samples, $R_{pre}^{2}$ and RMSEP were 0.86 and 0.577 °Brix, respectively (Figure 10e). The performance of the SSC prediction model was excellent in the order of Distances 1 and 2, and the optimal preprocessing conditions were range normalization and mean normalization.

Finally, at Level VI, the SSC prediction model that applied the SNV under Distance 1 showed the best performance (Table 11). By using this predictive model, $R_{cal}^{2}$, RMSEC, and the factor were 0.98, 0.154, and 15, respectively, and $R_{pre}^{2}$ and RMSEP were 0.89 and 0.596 °Brix, respectively, in predicting with unknown samples. Figure 10f shows the performance of the SSC prediction model was excellent in the order of Distances 3 and 2, and the optimal preprocessing conditions were when mean normalization and MSC were applied.

As a result of developing the SSC prediction model considering the size of the apple, MSC and SNV were the best preprocessing methods overall, and the higher the level, the better the SSC prediction accuracy. In addition, the results of this study show better performance than those of the study that did not consider changes in the distance between the light source and the Vis/NIR sensor for the fruit size. The results of this study show better performance than those of predicting the SSC of apples using the Vis/NIR (400–1100 nm) spectrum as a reflection method ($R_{pre}^{2}$ = 0.82 and RMSEP = 0.5766) [22]. In the reflective method, stray light is generated, and transmittance spectroscopy appears to yield better results because it does not penetrate the entire fruit. [19]. In predicting SSC by measuring the spectrum excluding the center of the “Fuji” apple by developing an online transmittance device using Vis/NIR, the study showed better results with $R_{pre}^{2}$ at 0.733 and RMSEP at 0.61% [20]. In a study predicting the SSC of apples through an online semi-transmittance device using NIR, apples were divided into three stages considering only the diameter, and SSC was predicted using the diameter. The results showed that the study performed better when the apple diameter was 65–75 mm (similar in size to Level VI in this experiment), with 0.886 for R_pre_ and 0.536% for RMSPE [23]. This study shows that the SSC prediction performance may vary depending on the difference in the diameter of the fruit when the positions of the light source and spectroscopic sensor are fixed.

This study showed better results than those of previous studies. These show that in apple transmission spectroscopy, adjusting the positions of the light source and NIR sensor depending on the size of the apple has a significant impact on SSC prediction performance. In addition, it showed better performance in Levels IV–VI than in Levels I–III because the amount of light transmitted increased as the size of the apple decreased, resulting in higher signals. In the future, the development of an online Vis/NIR transmittance spectroscopy device that changes the position of the light source and Vis/NIR sensor according to the size of the apple and strengthens the light source will enable the development of a fast and high-performance SSC sorter. In addition, if the distance between the light source and Vis/NIR sensor is quickly adjusted based on the size of the apple, it is expected that a combination of one light source and one sensor can develop a non-destructive SSC sorter with higher performance than before for apples of various sizes. However, this study was conducted on the Fuji cultivars of apples, and additional research is needed to verify if it can be applied to various varieties as well.

## 4. Conclusions

In this study, the optimal distance between the light source and the Vis/NIR sensor that was not significantly affected by size was investigated, and the best SSC prediction models using PLSR for the determined distance were developed. The three measurement conditions under which the Vis/NIR signal was less affected by the apple size were 125 and 75 mm (Distance 1), 135 and 75 mm (Distance 2), and 135 and 80 mm (Distance 3). The SSC prediction models were developed by applying PLSR to the three measurement conditions for each apple size, and various preprocessing methods were applied to improve the performance of the SSC prediction model. Optimal results were obtained when Levels I, III, and VI were Distance 1; Levels IV and V were Distance 3; and Level 2 was Distance 2. The best performance of these was the measurement of Level 4 apples at Distance 3, and $R_{pre}^{2}$ was 0.91° while RMSEP was 0.508 °Brix when MSC was applied. The results of this study demonstrate the possibility of improving the SSC measurement performance of apples by adjusting the distance between the light source and the spectral sensor based on the size of the fruit. In the future, research will be conducted to simultaneously apply a shape detection system that uses not only the weight of the apple but also its diameter and height, as well as a distance control system between the light source and the spectral sensor based on the size of the apple.

## Figures and Tables

**Table 1:** Size classification of apples in standard specifications of agricultural products.

Level	3	4	5	6	7	8
Weight (g)	375 ≥	300 ≥	250 ≥	214 ≥	188 ≥	167 ≥
		375 <	300 <	250 <	214 <	188 <

**Table 2:** Characteristics of apple samples.

		Level					
		I	II	III	IV	V	VI
Experiment 1	Number of samples (n)	3	3	3	3	3	-
	Average weight (g)	390	325	292	240	197	-
	Average of maximum diameter (mm)	106.59	103.32	94.43	86.74	80.71	-
	Average height (mm)	84.99	82.37	75.33	73.2	66.1	-
Experiment 2	Number of samples (n)	82	57	72	60	70	70
	Average weight (g)	398	318	285	223	195	182
	Average of maximum diameter (mm)	99.16	90.17	88.23	80.80	78.92	75.63
	Average height (mm)	86.78	83.07	79.64	73.96	70.32	68.90

**Table 3:** Distance range between light source and apple and between apple and Vis/NIR sensor.

	Range I	Range II		Range III		Full Range
Distance between light and apple (mm)	60–80	70–90		80–100		60–100
	**Range ii**		**Range ii**		**Full Range**	
Distance between apple and Vis/NIR sensor (mm)	20–35		25–40		20–40	

**Table 4:** Average CV (%) for each distance range between light and apple and between apple and Vis/NIR sensor.

Level I–V CV (%) Average (Ranking)		Distance between Apple and NIR Sensor (mm)		
		Range i	Range ii	Full Range
		(20–35)	(25–40)	(20–40)
Distance between light and apple (mm)	Range I	5.275	5.057	5.160
	(60–80)	(9)	(6)	(8)
	Range II	4.806	5.025	5.150
	(70–90)	(3)	(5)	(7)
	Range III	4.457	4.760	5.022
	(80–100)	(1)	(2)	(4)
	Full Range	5.764	5.651	5.799
	(60–100)	(11)	(10)	(12)

**Table 5:** The number and SSC distribution of apple samples for each level.

		Level					
		I	II	III	IV	V	VI
Number of samples (Dataset)	Calibration	58	40	51	42	49	49
	Prediction	24	17	21	18	21	21
	Total	82	57	72	60	70	70
Number of Spectra	Calibration	232	160	204	168	196	196
	Prediction	96	68	84	72	84	84
	Total	328	228	288	240	280	280
SSC (°Brix)	Avg. ^1^	15.10	14.93	14.38	13.68	14.57	13.86
	SD ^2^	1.31	1.06	1.80	1.77	1.51	1.21

^1^ Avg.: average; ^2^ SD: standard deviation.

**Table 6:** Level I—the best PLSR model results of predicting SSC of apples for three optimal distances between the light source and the Vis/NIR sensor.

Level I	Preprocessing	Factor	Calibration		Prediction	
Cal.: 58, Pre.: 24			$R_{cal}^{2}$	RMSEC (°Brix)	$R_{pre}^{2}$	RMSEV (°Brix)
Distance 1	MSC	10	0.90	0.414	0.68	0.769
Distance 2	SNV	11	0.90	0.413	0.61	0.861
Distance 3	SNV	11	0.81	0.562	0.58	0.895

**Table 7:** Level II—the best PLSR model results of predicting SSC of apples for three optimal distances between the light source and the Vis/NIR sensor.

**Level II**	**Preprocessing**	**Factor**	**Calibration**		**Prediction**	
**Cal.: 40, Pre.: 17**			$R_{cal}^{2}$	**RMSEC** **(** **°Brix)**	$R_{pre}^{2}$	**RMSEV** **(°Brix)**
Distance 1	Raw	13	0.97	0.202	0.70	0.619
Distance 2	Raw	13	0.96	0.223	0.72	0.615
Distance 3	Raw	11	0.93	0.301	0.66	0.738

**Table 8:** Level III—the best PLSR model results of predicting SSC of apples for three optimal distances between the light source and the Vis/NIR sensor.

Level III	Preprocessing	Factor	Calibration		Prediction	
Cal.: 51, Pre.: 21			$R_{cal}^{2}$	RMSEC (°Brix)	$R_{pre}^{2}$	RMSEV (°Brix)
Distance 1	SNV	15	0.99	0.142	0.74	0.822
Distance 2	Normalization (Mean)	12	0.96	0.358	0.72	0.851
Distance 3	SNV	12	0.96	0.398	0.71	0.919

**Table 9:** Level IV—the best PLSR model results of predicting SSC of apples for three optimal distances between the light source and the Vis/NIR sensor.

Level IV	Preprocessing	Factor	Calibration		Prediction	
Cal.: 42, Pre.: 18			$R_{cal}^{2}$	RMSEC (°Brix)	$R_{pre}^{2}$	RMSEV (°Brix)
Distance 1	Normalization (Maximum)	11	0.97	0.319	0.85	0.705
Distance 2	Normalization (Range)	11	0.93	0.467	0.86	0.772
Distance 3	MSC	12	0.99	0.195	0.91	0.508

**Table 10:** Level V—the best PLSR model results of predicting SSC of apples for three optimal distances between the light source and the NIR sensor.

Level V	Preprocessing	Factor	Calibration		Prediction	
Cal.: 49, Pre.: 21			$R_{cal}^{2}$	RMSEC (°Brix)	$R_{pre}^{2}$	RMSEV (°Brix)
Distance 1	Normalization (Range)	14	0.97	0.251	0.80	0.730
Distance 2	Normalization (Mean)	14	0.98	0.199	0.76	0.784
Distance 3	MSC	11	0.90	0.487	0.86	0.577

**Table 11:** Level VI—the best PLSR model results of predicting SSC of apples for three optimal distances between the light source and the NIR sensor.

Level VI	Preprocessing	Factor	Calibration		Prediction	
Cal.: 49, Pre.: 21			$R_{cal}^{2}$	RMSEC (°Brix)	$R_{pre}^{2}$	RMSEV (°Brix)
Distance 1	SNV	15	0.98	0.154	0.89	0.596
Distance 2	MSC	11	0.90	0.337	0.76	0.841
Distance 3	Normalization (Mean)	13	0.93	0.275	0.85	0.742

## Data Availability

Data are contained within the article.
